# Fibrinogen Fucosylation as a Prognostic Marker of End-Stage Renal Disease in Patients on Peritoneal Dialysis

**DOI:** 10.3390/biom10081165

**Published:** 2020-08-09

**Authors:** Marko Baralić, Nikola Gligorijević, Voin Brković, Jaroslav Katrlík, Lucia Pažitná, Miloš Šunderić, Goran Miljuš, Ana Penezić, Zorana Dobrijević, Mirjana Laušević, Olgica Nedić, Dragana Robajac

**Affiliations:** 1 Department of Nephrology, Clinical Centre of Serbia, 11000 Belgrade, Serbia; baralicmarko@yahoo.com (M.B.); voin.brkovic@gmail.com (V.B.); mlausevic@gmail.com (M.L.); 2 Department of Metabolism, Institute for the Application of Nuclear Energy (INEP), University of Belgrade, 11080 Belgrade, Serbia; nikolag@inep.co.rs (N.G.); milos@inep.co.rs (M.Š.); goranm@inep.co.rs (G.M.); anap@inep.co.rs (A.P.); zorana.dobrijevic@inep.co.rs (Z.D.); olgica@inep.co.rs (O.N.); 3 Institute of Chemistry, Slovak Academy of Sciences, 84538 Bratislava, Slovakia; katrlik@yahoo.com (J.K.); pazitna.lucia@gmail.com (L.P.); 4 School of Medicine, University of Belgrade, 11000 Belgrade, Serbia

**Keywords:** ESRD, PD, carbohydrates, fibrinogen chains, lectin-based microarray

## Abstract

Glycosylation may strongly affect protein structure and functions. A high risk of cardiovascular complications seen in patients with end-stage renal disease (ESRD) is, at least partly associated with delayed clot formation, increased clot strength, and delayed cloth lysis. Taking into consideration that fibrinogen mediates these processes, we isolated fibrinogen from the plasma from patients with ESRD on peritoneal dialysis (ESRD-PD), and examined glycosylation of native fibrinogen and its subunits by lectin-based microarray and lectin blotting. Compared to healthy controls, fibrinogen from patients had increased levels of A2BG2 and decreased levels of FA2 glycan. The distribution of glycans on individual chains was also affected, with the γ chain, responsible for physiological functions of fibrinogen (such as coagulation and platelet aggregation), being most prone to these alterations. Increased levels of multi-antennary N-glycans in ESRD-PD patients were also associated with the type of dialysis solutions, whereas an increase in the fucosylation levels was strongly related to the peritoneal membrane damage. Consequently, investigation of fibrinogen glycans can offer better insight into fibrinogen-related complications observed in ESRD-PD patients and, additionally, contribute to prognosis, choice of personalised therapy, determination of peritoneal membrane damage, and the length of utilization of peritoneum for dialysis.

## 1. Introduction

Glycoconjugates are complex structures formed after enzyme-catalysed binding of carbohydrates to proteins, lipids, or nucleic acids [1]. Changes in the content of fucose (Fuc), bisecting N-acetylglucosamine (GlcNAc), sialic acid (Sia), branching, and (pauci)mannosidic structures are reported in various pathological conditions, and some glycoproteins are already recognised as diagnostic biomarkers (e.g., CEA, MUC1, MUC16, CA19-9, PSA) [1]. Lectins are often used to assess the glycan content, as they recognise and interact with individual mono/oligosaccharides or more complex structures (e.g., glycans). These interactions depend on the lectin structure, its binding site and organization, and lectin multivalence [2]. A variety of different lectin-based techniques are employed in glycoprofiling, as they are affordable, fast, and high-throughput [2].

Glycoconjugates are inevitable actors in the kidney functioning, especially in glomerular filtration [1]. Peritoneal dialysis (PD) is a type of treatment for some patients with end-stage renal disease (ESRD) in which the peritoneum is used as a natural membrane through which osmosis and diffusion take place, eliminating the harmful products of metabolism and excess fluid. However, this procedure can contribute to the appearance of peritonitis and peritoneal membrane damage, as well as systemic inflammation and endothelial dysfunction [3]. Peritonitis and the loss of mesothelial cell mass correlate with an increase in the content of IgG-related glycans present in peritoneal effluent samples and a decrease in the galactosylation of biantennary glycans [4]. Additionally, patients with chronic kidney disease (CKD) are at higher risk of cardiovascular morbidity and mortality [5]. Delayed clot formation, decreased lysis, and increased clot strength, as well as hypercoagulability found in patients with CKD, are to some extent mediated by fibrinogen [6]. This key component of the blood clotting process is a 340 kDa homodimer, consisting of two sets of three polypeptide chains (AαBβγ)_2_ that are O- and/or N-glycosylated. The Aα chain possesses two potential N-glycosylation sites, with glycosylation of Asn686 being reported just recently, whereas the Ββ chain is N-glycosylated at Asn394 and γ chain at Asn78 [7]. Fibrinogen is abundantly O-glycosylated at various sites on the Aα chain and only at one site on the Bβ chain [7,8].

Altered glycosylation affects fibrinogen properties in hepatic diseases [9,10] and the present study aimed to examine fibrinogen glycosylation in patients with ESRD-PD, as that may help in better understanding of the mechanisms underlying fibrinogen-related changes, and possibly assist in the therapeutic approach. Lectin-based protein microarray and lectin blotting were applied to define glycan structures on fibrinogen.

## 2. Experimental Section

### 2.1. Blood Samples

Blood samples from patients on PD were collected at the Clinical Centre of Serbia. The total number of patients treated for PD in the centre was 80, but 28 of them met one or more exclusion criteria, leaving a total of 52 patients eligible for the current study. The exclusion criteria were: less than six months on PD treatment, infection of the peritoneal catheter exit site, presence of acute peritonitis three months before sampling, administration of antiplatelet and anticoagulant therapy six months before blood sampling, known coagulopathy and haematological malignancy, acute or chronic liver damage, and presence of anti-HCV, HbsAg, and HIV 1/2. All patients selected for the study underwent a continuous ambulatory PD treatment using 1.36% and/or 2.25% glucose solutions, while six of them used icodextrin for the longest dialysis shift. None of the patients used heparin intraperitoneally. In addition to their regular therapy and depending on their individual needs, patients also received calcium carbonate, vitamin D, erythropoietin, angiotensin II receptor blocker, angiotensin-converting enzyme inhibitor, and calcium channel blocker. Blood from healthy persons, used as control samples (HC, *n* = 32), was collected at INEP. Clinical and biochemical parameters of patients and adequate healthy controls are presented in Table 1, whereas the details of continuous ambulatory peritoneal dialysis (CAPD) are shown in Table 2. Informed consent was obtained from each participant involved in the study. The study was approved by the Ethical Committees of INEP and the Clinical Centre of Serbia (approval number: 890/8) and conducted following the Declaration of Helsinki and the Ethical Guidelines for Medical and Health Research Involving Human Subjects.

### 2.2. Fibrinogen Isolation

Blood samples, collected in tubes with EDTA as an anticoagulant, were centrifuged at 800 *g* for 5 min. The obtained supernatant (blood plasma) was treated with a saturated ammonium-sulphate (AS) solution to the final saturation of AS of 20% and centrifuged 5 min at 10,000 *g*. The precipitate was washed in 20% AS in 50 mM PBS and centrifuged. The final precipitate (representing isolated fibrinogen) was dissolved in 50 mM PBS and stored at −20 °C.

### 2.3. Lectin-Based Protein Microarray

Fibrinogen samples, diluted in 50 mM PBS pH 7.4 to 100 μg/mL, were printed onto microarray slides coated with epoxysilane (NEXTERION Slide E, Schott, Germany), in triplicate into eight identical subarrays using a non-contact piezoelectric printer sciFLEXARRAYER S1 and piezo dispense capillary PDC 90 (Scienion AG, Berlin, Germany), at a temperature of 14 °C and humidity of 60%, and incubated at 4 °C for 2 h. Unoccupied epoxy groups were blocked with 3% BSA in PBS, at 4 °C for 1 h. After washing, printed proteins were incubated with biotinylated lectins (Table 3) at the concentration of 25 µg/mL in PBS with 0.05% Tween 20 (PBST), at 25 °C for 1 h. All lectins were purchased (Vector, Burlingame, CA, USA) except PhoSL which was a kind gift from Dr. Yuka Kobayashi (J-Oil Mills Inc., Yokohama, Japan). After thorough washing, bound lectins were allowed to interact with 0.5 µg/mL CF647-streptavidin conjugate (Biotium, Hayward, CA, USA) in PBST at 25 °C for 15 min. Slides were further thoroughly washed with PBST and distilled water, dried by centrifugation, and scanned using the InnoScan^®^ 710 fluorescent scanner (Innopsys, Carbonne, France). Fluorescent signals were analysed using the Mapix^®^ 5.5.0 software (Innopsys).

### 2.4. Lectin Blotting

Isolated fibrinogen samples were grouped into 16 PD and nine HC pools and subjected to reducing SDS-PAGE (10% gel). Resolved fibrinogen chains were transferred to a nitrocellulose membrane, stained with Ponceau S, and incubated with biotinylated lectins (Table 3). HRP-conjugated avidin D (Vector, Burlingame, USA) and the ECL reagent (Pierce Biotechnology, Rockford, IL, USA) were used for protein visualisation by autoradiography. Densitometric analysis of the obtained signals was done using the TotalLab software (Amersham BioSciences, Buckinghamshire, UK), and the values were normalised against the signals obtained after the Ponceau S staining.

### 2.5. Statistics

Comparisons between the two groups of samples (PD vs. HC) were performed using the non-parametric Mann-Whitney U test. Differences in categorical variables were tested by the χ^2^ test or Fisher test, as appropriate. The Pearson linear correlation coefficient was used to analyse the relationships between dialysis vintage and fibrinogen glycosylation. In the PD group, multivariate (forward Wald) binary logistic regression analysis was employed to evaluate the relationship between the outcome variable (ultrafiltration rate more or less than 700 mL per day) and lectins as potential determinants adjusted for residual diuresis. The discriminatory power of the model was assessed using the receiver operating characteristic (ROC) curve. Statistical analyses were performed using SPSS v.18 (Chicago, IL, USA). Statistical significance was defined as *p* < 0.05.

## 3. Results

### 3.1. Samples

Clinical and biochemical characteristics of patients and healthy controls are given in Table 1, while the details of CAPD are given in Table 2. Distribution of male and female patients was the same in both investigated groups (Table 1). Compared with the HC group, patients on PD had lower levels of total blood proteins (as well as albumin) and iron, and considerably higher levels of urea, creatinine, fibrinogen, and sedimentation rate, as was expected due to kidney failure and systemic inflammation.

### 3.2. Lectin-Based Microarray

The choice of lectins was made based on the previously reported data on glycosylation of fibrinogen and other abundant serum glycoproteins (Table 3). Six out of sixteen employed lectins gave signals with the signal-to-noise ratios S/N ≤ 10, whereas ten gave signals with S/N > 10 (Table 3).

PNA and MAL-I interacted with S/N < 3 and were, therefore, omitted from further investigations. Both PD and HC fibrinogen molecules interacted with the same lectins, pointing to the presence of the same type of glycan residues: Biantennary N-glycans (ConA and PHA-E) terminating with α2,6-Sia bound to Gal/GalNAc or GlcNAc (SNA and WGA) and/or Gal (RCA), with bisecting GlcNAc (PHA-E) and/or α1,6-Fuc (core-Fuc) (LCA, AAL, and PhoSL) and or α1,3-Fuc (antennary Fuc) (AAL). A low presence of multi-antennary N-glycans terminating with Gal (PHA-L) was also found. Signals obtained with ConA, NPL, and GNL pointed to the possible presence of high-mannose (Man) N-glycans. SNA, RCA, GSL-I, and WGA signals also indicated the presence of O-glycans of T and Tn antigen type (Galβ1,3GalNAcα1-O-Ser/Thr and GalNAcα1-O-Ser/Thr) terminating, to some extent, with Sia (sialyl-T(n)) (SNA and MAL-II).

**Figure 1:**
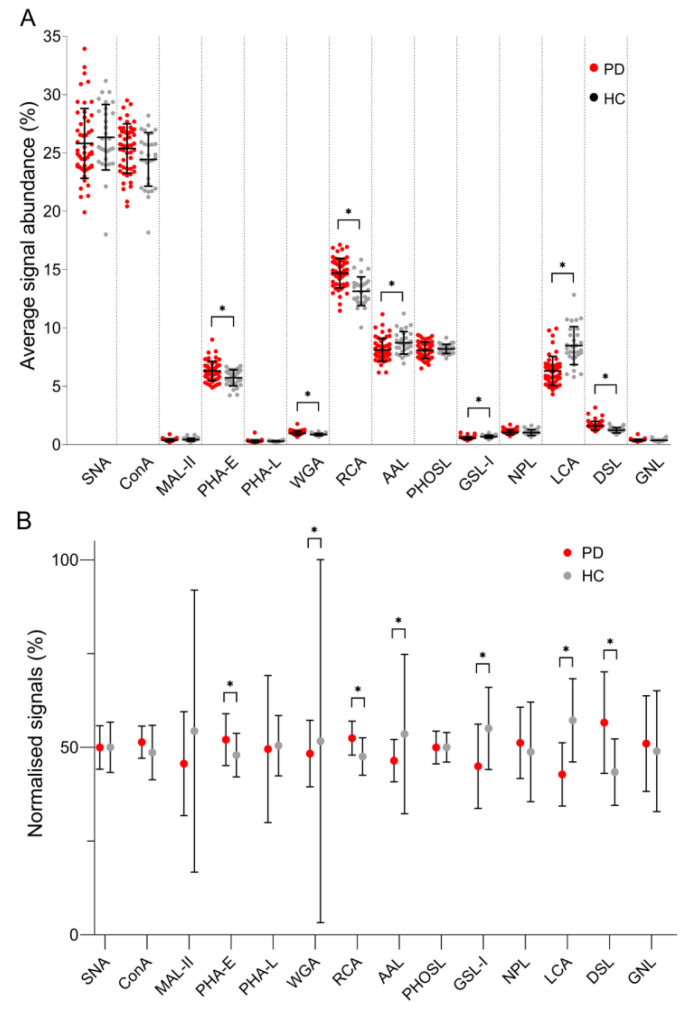
(**A**) Relative signal abundances obtained for each lectin used in the lectin-based microarray analysis of fibrinogen samples, (**B**) the contribution of each group (PD and HC) calculated as the portion of the sum of signals obtained for each lectin. ESRD patients on peritoneal dialysis (PD), healthy controls (HC). Relative abundances are given together with standard deviation, while the Mann-Whitney U test *p*-value < 0.05 is labelled with “*”.

To acquire information on the impact of ESRD-PD on the distribution of fibrinogen glycans, signals obtained for all lectins were summed up for each individual sample and the percentage of each lectin-specific signal was further presented as its relative abundance (Figure 1A). In further data processing, signals originating from each individual lectin were summed up for two groups of samples, the sum was defined as 100%, and the contribution of each group (PD and HC) was calculated as the portion of that sum (Figure 1B).

In fibrinogen from the PD group, the content of glycans recognised by PHA-E, RCA, and DSL was increased, whereas the content of glycans recognised by WGA, AAL, GSL-I, and LCA was decreased. The observed changes implied that, due to a kidney failure, the content of A2BG2 (PHA-E binding, Figure S1) and glycans with GlcNAc and/or Gal residues with or without Sia (DSL and RCA) increase. DSL binding suggested an increase in the content of multi-antennary N-glycans. The content of fucosylated glycans (AAL binding), FA2 (LCA binding), and N-glycans with GlcNAc residues decorated with Sia (WGA) decreased due to pathology. DSL recognises β1,4-Gal in N-glycans, unlike GSL-I that binds β1,3-Gal in the T type of O-glycans, whose content in this pathology also decreased together with the content of T type O-glycans (WGA).

**Figure 2:**
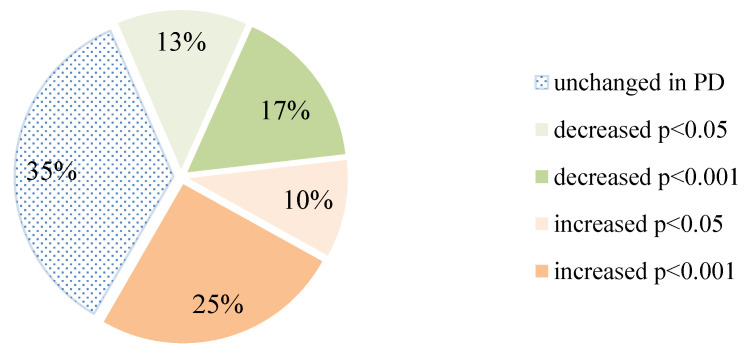
Distribution of the calculated lectin/lectin ratios based on their statistical significance.

To acquire additional information on ESRD-induced changes in patients on PD, signals obtained for each of the 14 lectins were compared, their ratios calculated, and statistically analysed (Table 4). Out of all ratios, two-thirds were changed in the PD group, while decreasing and increasing events were almost evenly distributed (Figure 2). Although the significance was set at *p* < 0.05, for more than two-thirds of the results which exhibited an increase in the PD group, this significance was even lower than 0.001. It is worth noting that when the reactivity of other lectins was compared to the reactivity of LCA and DSL, they all exhibited an increased binding when compared to LCA and a decrease in the case of DSL in the PD patient.

#### Association between Lectin-Based Microarray Results and Clinical Data

Although there is a possibility that protein (i.e., fibrinogen) glycosylation can be affected by the composition of dialysis solutions, a more conclusive discussion cannot be offered, due to a small number of patients (*n* = 6) receiving glucose polymer (icodextrin) for the longest dialysis shift. Even so, the analysis was performed and statistical difference was found in the case of PHA-L lectin, showing that patients treated with icodextrin had a higher content of multi-antennary N-glycans terminating with Gal compared to patients treated with 1.36% (*p* = 0.010) or 2.25% glucose solution (*p* = 0.007). Dialysis vintage did not affect the obtained results (*p* = 0.337).

The data on fibrinogen glycosylation were further correlated with the parameters of kidney function and peritoneal membrane status, to examine whether they can be used as a diagnostic/prognostic marker. Anuric patients (with residual urine levels less than 100 mL per day) did not differ from the rest of the group. The samples were further organised based on the ultrafiltration rate with the cut off set at 700 mL. Lectin signals were related to the ultrafiltration rate, adjusted for the residual diuresis values, and results presented in Table 5.

**Figure 3:**
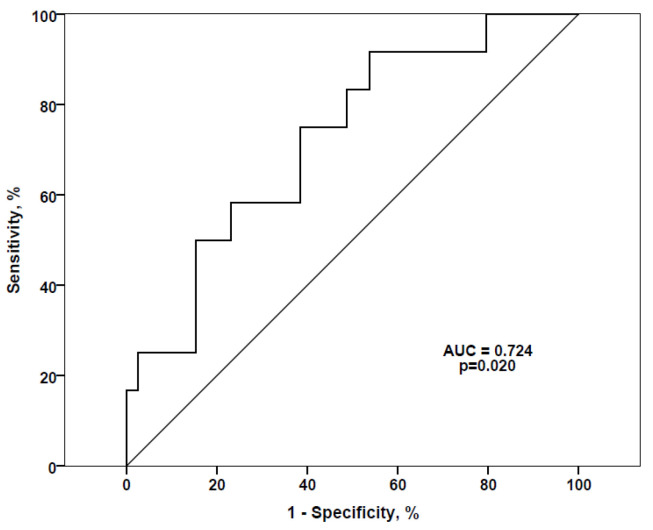
Receiver operating characteristic (ROC) curve for the antennary Fuc (AAL) as a predictor of ultrafiltration rate. AUC: Area under the curve.

NPL, AAL, DSL, GSL-I, LCA, and MAL-II lectins profiled as independent predictors of the ultrafiltration rate. Higher signal values obtained for these lectins were related to lower ultrafiltration capacity, further indicating higher damage of the peritoneal membrane. The discriminatory power of the model was assessed using the receiver operating characteristic (ROC) curve (Figure 3). Although signals for all lectins were examined, the significance was found only for those obtained with the AAL lectin, with AUC of 72.4%, signifying the importance of fibrinogen fucosylation as the potential predictor/marker of the peritoneal membrane damage.

### 3.3. Lectin Blotting

**Figure 4:**
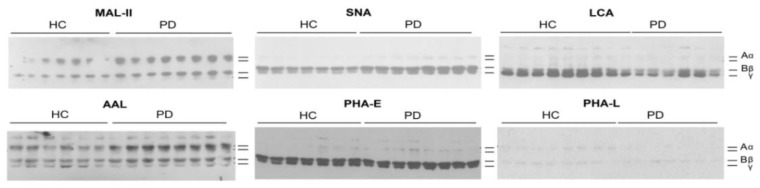
Lectin blots of fibrinogen samples resolved under reducing SDS-PAGE and incubated with biotinylated lectins. ESRD patients on peritoneal dialysis (PD) and healthy controls (HC). Positions of fibrinogen chains are marked on the right.

To investigate whether ESRD affects the distribution of glycans on individual fibrinogen chains, isolated fibrinogen samples were subjected to reducing SDS-PAGE and further incubated with biotinylated lectins. The choice of lectins was based on the data obtained with microarray and with the focus on the presence of multi-antennary, fucosylated, and sialylated N-glycans. The presence of the same type of glycans in two groups of investigated samples was again confirmed (Figure 4). Interaction with PHA-L was very weak, suggesting a relatively low content of multi-antennary N-glycans. When signals originating from individual chains were summed-up and analysed, statistical significance was found only in the case of LCA (*p* = 0.0026), indicating that the content of FA2 glycan decreased due to ESRD. These results are in accordance with those obtained by the lectin-based microarray.

When glycosylation of individual fibrinogen chains was examined, statistical significance between two groups of samples was found as shown in Table 6.

In contrast to the Aα chain, the glycan content of the Bβ and γ chains was affected by ESRD-PD. The amounts of α2,3/6-sialyl-T/Tn type of O-glycans (reactive with MAL-II) on the Bβ chain and FA2 N-glycan on the γ chain (LCA) increased in the ESRD-PD. On the other hand, the total α2,6-Sia bound to Gal of N-glycans (SNA) and antennary and/or core-fucosylated glycans (AAL) on both β and γ chains decreased, while the content of A2BG2 decreased only on the γ chain (PHA-E). The ratios Aα/γ and Bβ/γ increased in pathology regarding the content of fucosylated glycans (AAL), as well as the Aα/γ ratio regarding the content of A2BG2 (PHA-E). On the contrary, the Bβ/γ ratio decreased in the case of FA2 N-glycan (LCA).

## 4. Discussion

Patients with ESRD are prone to cardiovascular complications, and one of the contributing factors is the formation of thicker clots that are more resistant to fibrinolysis [5,6]. The focus of the current study was set on the fibrinogen structure-related changes in ESRD patients on PD. We investigated glycosylation of the native form of fibrinogen and its individual chains by employing the lectin-based microarray and lectin blotting. Lectin-based microarrays are very useful in determining the accessibility of specific glycan groups without a prior release of glycans or glycan purification, giving information on native glycoproteins, being suitable for the first-line screening of different biological samples [11]. Fibrinogen is rich in A2G2S1 and A2G2S2 glycans, while the same structures with bisecting GlcNAc or Fuc and triantennary structures are less abundant [7,12,13]. At the same time, Aα and Bβ chains are also heavily O-glycosylated bearing rather (sialyl)T than (sialyl)Tn type glycans [8,13,14,15].

In general, the literature data on protein glycosylation in kidney disease relies only on a few studies, with complete absence on information regarding fibrinogen glycosylation. While the levels of α2,6-Sia were higher in patients with early-stage diabetic kidney disease [16], examination of the relative abundance of Galβ1,3GalNAc was proposed for differentiating diabetic and non-diabetic nephropathy [17]. Increased levels of plasma A2BG1S1, FA2G2S1, and FA2G2S2 were associated with CKD, while FA2G2S1 originating from IgG was found increased in CKD patients [18,19]. A lower risk of CKD was observed in individuals with galactosylated IgG and IgG with sialylated and core-fucosylated glycans IgG, while patients possessing more agalactosylated IgG and IgG with bisecting GlcNAc were under an increased risk of CKD [19]. The higher survival rate in patients on haemodialysis was related to the overall lower content of serum triantennary glycan—FA3G3S2 [20], whereas thinner fibrin fibres found in these patients resulted, at least partly, from fibrinogen guanidinylation and glycosylation [21]. In ESRD patients on PD, increase in the sialylation of multi-antennary N-glycans of peritoneal effluent proteins was related to a negative outcome, while peritonitis and the loss of mesothelial cell mass were associated with an increase in the levels of presumably IgG-related glycans and a decrease of galactosylation of biantennary glycans [4].

Our findings imply that, due to a kidney pathology, the content of A2BG2 increases whereas the content of FA2 decreases on the fibrinogen molecule, as was also reported for IgG glycans [19]. Fucosylation is involved in various processes including cell adhesion, proliferation, tissue development, angiogenesis, fertilization, tumour development and metastasis, and altered fucosylation was observed in numerous inflammatory processes [22]. An increase in antennary fucosylation of α_1_-acid glycoprotein was related with vascular complications in patients with type 1 diabetes [23]. In patients with type 2 diabetes, a faster decline of kidney functions was associated with IgG glycans containing more bisecting GlcNAc and increased fucosylation with bisecting GlcNAc, whereas fucosylation without bisecting GlcNAc was associated with a slower kidney deterioration [24]. Inhibition of core-fucosylation alleviated kidney damage and fibrosis in a diabetic mouse model [25]. According to our experimental results, with AUC of 72.4%, a degree of fucosylation of fibrinogen seems to be strongly related to the loss of peritoneal membrane function.

We also found an increase in the content of multi-antennary N-glycans, which was seen on the entire serum N-glycome as well [26], when it was suggested that these glycans may be part of the mechanism of diabetic kidney disease development. Although the dialysis vintage did not affect the obtained results, there was a significant difference in the content of multi-antennary N-glycans in respect to the solutions used for PD. Compared to patients receiving a glucose solution, patients receiving a glucose polymer for the longest dialysis shift had a higher content of multi-antennary N-glycans. Although these data have to be taken with caution, due to a low number of patients under such treatment involved in the present study and no information on the survival rate, this finding seems interesting and a logical direction for future investigations in terms of protein glycosylation in ESRD. More complex N-glycan structures were found in patients with higher glycaemia and steeper decline in the estimated glomerular filtration rate [26]. In diabetic patients, a higher efflux of glucose occurs, and due to the available sugar source, an increase in complex N-glycans in these patients is expected [26]. One can speculate that the same may occur in patients on PD, due to long exposures to high concentrations of glucose solutions [27].

As expected, fibrinogen glycosylation to some extent follows the general pattern of serum N-glycosylation. The γ chain that is responsible for fibrinogen interaction with the cell surface receptors, as well as binding of growth and coagulation factors, enables clot formation, platelet aggregation, and wound healing [28]. The distribution of glycans on individual fibrinogen chains was altered, with the γ chain being most susceptible to alterations, thus, to some extent explaining fibrinogen-related changes present in the ESRD patients. It was reported that liver cirrhosis lowers the content of O-glycans present on the Aα chain, while the content of Sia and Fuc of N-glycans present on the Bβ and γ chain increases due to liver cirrhosis and hepatocellular carcinoma [9,10]. Our findings are somewhat different. We did not observe significant changes in the glycan content of the Aα chain and the content of the Bβ and γ chain glycans containing Sia and Fuc decreased. Only the content of fucosylated biantennary glycan (FA2) on the γ chain increased in the samples from ESRD-PD. Both liver cirrhosis and ESRD are characterised by fibrosis of the affected organs, but these conditions modify fibrinogen glycans differently.

## 5. Conclusions

We report on the considerable and statistically significant alterations in the glycosylation pattern of fibrinogen that occur in ESRD patients on PD. Most susceptible to these changes is the γ chain, which is responsible for physiological functions of fibrinogen such as coagulation and platelet aggregation. Additionally, since an increase in fibrinogen fucosylation is related to the loss of the membrane function, it seems that more thorough investigations might be a good basis for finding a potential diagnostic and prognostic marker. Thus, information on fibrinogen glycans may help in clarifying some aspects of ESRD, including possible cardiovascular complications, and can also assist in defining the length of utilization of peritoneum for dialysis.

## Figures and Tables

**Table 1:** Clinical and biochemical characteristics of end-stage renal disease (ESRD) patients on peritoneal dialysis (PD) and healthy control persons (HC). Data are presented as the median ± interquartile range. The statistically significant difference between the groups (*p* < 0.05) is labelled with “*”.

	Peritoneal Dialysis (PD), *n* = 52		Healthy Control (HC), *n* = 32	
	Male, *n* = 26	Female, *n* = 26	Male, *n* = 16	Female, *n* = 16
Age (years)	65.5 ± 15.8		61.0 ± 29.3	
Period on PD (months)	30.0 ± 49.5		/	
With peritonitis	20 (38.5%)		/	
With diabetes	20 (38.5%)		/	
Receiving erythropoietin	22 (42.3%)		/	
Ultrafiltration rate (mL/day)	1000 ± 650			
Residual urine (mL/day)	750 ± 975			
Biochemical parameters				
Glucose (mM)	5.6 ± 2.5		5.3 ± 0.9	
Urea (mM)	16.1 ± 6.3 *		5.1 ± 0.8	
Creatinine (μM)	653.0 ± 204.3 *		81.0 ± 22.0	
Uric acid (μM)	310.0 ± 62.5 *		325.0 ± 91.3	
Total protein (g/L)	65.0 ± 9.3 *		72.3 ± 2.7	
Albumin (g/L)	37.0 ± 6.0 *		47.5 ± 3.2	
Fibrinogen (g/L)	4.4 ± 0.9		2.7 ± 0.8	
Sedimentation	85.0 ± 44.0 *		13.0 ± 9.5	
Iron (mM)	11.5 ± 3.9 *		17.7 ± 3.8	

**Table 2:** Schedule of continuous ambulatory peritoneal dialysis (CAPD). Distribution of patients based on the concentrations of PD solution used and the number of dialysis exchange is given, in relation to the levels of residual urine (RU).

Glucose Solutions Used in CAPD	RU < 700 mL (*n* = 26)	RU > 700 mL (*n* = 26)
4 x * 1.36%	6	17
4 x 1.50%	6	3
3 x 1.36% + 1 x 2.27%	2	3
3 x 1.50% + 1 x 2.30%	2	1
2 x 1.36% + 2 x 2.27%	-	2
3 x 1.36% + 2 x 2.27%	4	-
2 x 1.36% + 2 x 2.27% + icodextrin	6	-

* x means time(s) per day.

**Table 3:** Carbohydrate specificities of lectins employed in the lectin-based microarray and lectin blot. Lectins used in each method are marked with “+”. For the lectin-based microarray, the obtained S/N ratios are given in brackets.

Lectin (Source)	Carbohydrate Specificity	Lectin Microarray	Lectin Blot
PNA (*Arachis hypogaea*)	Galβ1,3GalNAc	+ (S/N < 3)	
MAL-I (*Maackia amurensis*)	NeuNAcα2,3Galβ1,4GlcNAc	+ (S/N < 3)	
MAL-II (*Maackia amurensis*)	NeuNAcα2,3Galβ1,3(±NeuNAc2,6)GalNAc	+ (S/N < 10)	+
PHA-L (*Phaseolus vulgaris*)	Tri/tetraantennary complex type N-glycans w/terminal Gal	+ (S/N < 10)	+
DSL (*Datura stramonium*)	GlcNAcβ1,4GlcNAc oligomers; Galβ1,4GlcNAc	+(S/N < 10)	
GNL (*Galanthus nivalis*)	High mannose type N-glycans; Manα1,3Man	+ (S/N < 10)	
GSL-I (*Griffonia simplicifolia*)	Galα1,3Gal; Galα1,3GalNAc	+ (S/N < 50)	
WGA (*Triticum vulgaris*)	GlcNAcβ1,4GlcNAc; chitin oligomers; NeuAc	+ (S/N < 50)	
SNA (*Sambucus nigra*)	NeuNAcα2,6Gal/GalNAc	+ (S/N < 50)	+
NPL (*Narcissus pseudonarcissus*)	High mannose type N-glycans; Manα1,6Man	+ (S/N < 50)	
LCA (*Lens culinaris*)	αDGlc, αDMan in N-glycans with Fuca1,6GlcNAc	+ (S/N < 50)	+
PhoSL (*Pholiota squarrosa*)	Fucα1,6GlcNAc	+ (S/N < 50)	
PHA-E (*Phaseolus vulgaris*)	Galβ1,4GlcNAcβ1,2Man with bisecting GlcNAc	+ (S/N < 50)	+
AAL (*Aleuria aurantia*)	Fucα1,6GlcNAc; Fucα1,3(Galβ1,4)GlcNAc	+ (S/N < 50)	+
ConA (*Canavalia ensiformis*)	Manα1,6(Manα1,3)Man	+ (S/N > 50)	
RCA (*Ricinus communis*)	Galβ1,4GlcNAc	+ (S/N > 50)	

**Table 4:** Mann-Whitney U analysis of the lectin/lectin ratios of signals obtained using the lectin-based microarray.

vs.	SNA	ConA	MAL-II	PHA-E	PHA-L	WGA	RCA	AAL	PhoSL	GSL-I	NPL	LCA	DSL	GNL
SNA	1	0.02202	0.57548	0.01016	0.61708	0.0096	0.00104	0.26272	0.7417	0.00078	0.12114	0.00001	0.00001	0.29834
ConA		1	0.06576	0.11134	0.01878	0.13622	0.02642	0.0098	0.01242	0.00001	0.90448	0.00001	0.0007	0.56192
MAL-II			1	0.05	0.86502	0.00288	0.00362	0.88866	0.36812	0.06576	0.3843	0.00328	0.00001	0.30772
PHA-E				1	0.00001	0.77948	0.39532	0.00018	0.01732	0.00001	0.28014	0.00001	0.00188	0.67448
PHA-L					1	0.0001	0.00001	0.34722	0.12602	0.00012	0.09894	0.00001	0.00001	0.04236
WGA						1	0.53526	0.00001	0.00328	0.00001	0.02852	0.00001	0.00006	0.1031
RCA							1	0.00001	0.00001	0.00001	0.0278	0.00001	0.00152	0.30302
AAL								1	0.06576	0.00001	0.034	0.00001	0.00001	0.01552
PHOSL									1	0.00014	0.50286	0.00001	0.00001	0.29834
GSL-I										1	0.00018	0.0088	0.00001	0.00006
NPL											1	0.00001	0.00001	0.92034
LCA												1	0.00001	0.00001
DSL													1	0.00001
GNL														1
	SNA	ConA	MAL-II	PHA-E	PHA-L	WGA	RCA	AAL	PhoSL	GSL-I	NPL	LCA	DSL	GNL
0.05 > *p* > 0.001	0	4	1	2	0	4	4	0	0	1	1	0	2	2
*p* < 0.001	2	2	0	4	2	4	5	2	2	0	2	0	11	2
0.05 > *p* > 0.001	4	1	2	1	2	0	1	3	3	0	2	2	0	0
*p* < 0.001	1	1	1	0	4	1	0	4	2	11	1	11	0	1

Calculated “*p*” values; statistically significant data are labelled as follows: green color represents a decrease in patients on peritoneal dialysis (PD), red color represents an increase in patients on PD.

**Table 5:** Multivariate binary logistic regression analysis for the evaluation of the relationship between ultrafiltration rate and lectins adjusted for residual diuresis, with values obtained for PD samples.

	B	*p*	OR	CI 95%		B	*p*	OR	CI 95%
SNA		0.621	-	-	ConA	-	0.228	-	-
RD	−1.786	0.002	0.168	0.055–0.509	RD	−1.786	0.002	0.168	0.055–0.509
MAL-II	−0.074	0.045	0.929	0.864–0.998	PHA-E	-	0.323	-	-
RD	−2.006	0.001	0.134	0.040–0.451	RD	−1.786	0.002	0.168	0.055–0.509
PHA-L	-	0.536	-	-	WGA	-	0.068	-	-
RD	−1.786	0.002	0.168	0.055–0.509	RD	−1.786	0.002	0.168	0.055–0.509
RCA	-	0.213	-	-	AAL	−0.006	0.029	0.994	0.989–0.999
RD	−1.786	0.002	0.168	0.055–0.509	RD	−1.786	0.002	0.168	0.055–0.509
PhoSL	-	0.172	-	-	GSL-I	−0.044	0.037	0.957	0.918–0.997
RD	−1.786	0.002	0.168	0.055–0.509	RD	−1.949	0.001	0.142	0.043–0.471
NPL	−0.043	0.013	0.958	0.926–0.991	LCA	−0.005	0.039	0.995	0.991–0.999
RD	−2.084	0.002	0.124	0.034–0.456	RD	−1.959	0.001	0.141	0.043–0.466
DSL	−0.022	0.037	0.978	0.958–0.999	GNL	-	0.072	-	-
RD	−2.065	0.001	0.978	0.036–0.447	RD	−1.786	0.002	0.168	0.055–0.509

**Table 6:** Mann-Whitney U analysis of the lectin blot densitometric signals.

	MAL-II	SNA	LCA	AAL	PHA-E	PHA-L
Aα	0.27134	0.95216	-	0.22246	0.18352	0.07346
Bβ	0.01174 *	0.00214 *	0.06724	0.00652 *	0.3843	0.6818
γ	-	0.00932 *	0.00262 *	0.01278 *	0.03236 *	-
Aα/Bβ	0.65272	0.05614	-	0.86502	0.14706	0.18352
Aα/γ	-	0.27134	-	0.01278 *	0.03236 *	-
Bβ/γ	-	0.07346	0.00804 *	0.00318 *	0.52218	-

Calculated “*p*” values; statistically significant data are labelled as follows: green color represents a decrease in patients on peritoneal dialysis (PD), red color represents an increase in patients on PD.

## Supplementary Materials

The following are available online at https://www.mdpi.com/2218-273X/10/8/1165/s1. Figure S1: Structures of N- and O-glycans mentioned in the manuscript, with the carbohydrates annotation according to the recommendations of the Consortium for Functional Glycomics (CFG).

## References

[1] Reily C., Stewart T.J., Renfrow M.B., Novak J. (2019). Glycosylation in health and disease. Nat. Rev. Nephrol..

[2] Hendrickson O.D., Zherdev A.V. (2018). Analytical application of lectins. Crit. Rev. Anal. Chem..

[3] Kooman J.P., van der Sande F.M. (2019). Body fluids in end-stage renal disease: Statics and dynamics. Blood Purif..

[4] Ferrantelli E., Farhat K., Ederveen A.L.H., Reiding K.R., Beelen R.H.J., van Ittersum F.J., Wuhrer M., Dotz V. (2018). Effluent and serum protein N-glycosylation is associated with inflammation and peritoneal membrane transport characteristics in peritoneal dialysis patients. Sci. Rep..

[5] Schlieper G., Hess K., Floege J., Marx N. (2016). The vulnerable patient with chronic kidney disease. Nephrol. Dial. Transplant..

[6] Nunns G.R., Moore E.E., Chapman M.P., Moore H.B., Stettler G.R., Peltz E., Burlew C.C., Silliman C.C., Banerjee A., Sauaia A. (2017). The hypercoagulability paradox of chronic kidney disease: The role of fibrinogen. Am. J. Surg..

[7] Hoffmann M., Pioch M., Pralow A., Hennig R., Kottler R., Reichl U., Rapp E. (2018). The fine art of destruction: A guide to in-depth glycoproteomic analyses-exploiting the diagnostic potential of fragment ions. Proteomics.

[8] Zauner G., Hoffmann M., Rapp E., Koelman C.A.M., Dragan I., Deelder A.M., Wuhrer M., Hensbergen P.J. (2012). Glycoproteomic analysis of human fibrinogen reveals novel regions of O-glycosylation. J. Proteome Res..

[9] Nagel T., Klaus F., Ibanez I.G., Wege H., Lohse A., Meyer B. (2018). Fast and facile analysis of glycosylation and phosphorylation of fibrinogen from human plasma-correlation with liver cancer and liver cirrhosis. Anal. Bioanal. Chem..

[10] Gligorijević N., Minić S., Križáková M., Katrlík J., Nedić O. (2018). Structural changes of fibrinogen as a consequence of cirrhosis. Thromb. Res..

[11] Huang Y., Zhu H. (2017). Protein array-based approaches for biomarker discovery in cancer. Genom. Proteom. Bioinf..

[12] Adamczyk B., Struwe W.B., Ercan A., Nigrovic P.A., Rudd P.M. (2013). Characterization of fibrinogen glycosylation and its importance for serum/plasma N-glycome analysis. J. Proteome Res..

[13] Nagel T., Meyer B. (2014). Simultaneous characterization of sequence polymorphisms, glycosylation and phosphorylation of fibrinogen in a direct analysis by LC-MS. Biochim. Biophys. Acta.

[14] Pacchiarotta T., Hensbergen P.J., Wuhrer M., van Nieuwkoop C., Nevedomskaya E., Derks R.J., Schoenmaker B., Koeleman C.A.M., van Dissel J., Deelder A.M. (2012). Fibrinogen alpha chain O-glycopeptides as possible markers of urinary tract infection. J. Proteom..

[15] Hoffmann M., Marx K., Reichl U., Wuhrer M., Rapp E. (2016). Site-specific O-glycosylation analysis of human blood plasma proteins. Mol. Cell. Proteom..

[16] Mise K., Imamura M., Yamaguchi S., Teshigawara S., Tone A., Uchido H.A., Eguchi J., Nakatsuka A., Ogawa D., Yoshida M. (2018). Identification of novel urinary biomarkers for predicting renal prognosis in patients with type 2 diabetes by glycan profiling in a multicenter prospective cohort study: U-CARE study 1. Diabetes Care..

[17] Liu M., Yu H., Zhang D., Han Q., Yang X., Liu X., Wang J., Zhang K., Yang F., Cai G. (2018). Alteration of glycosylation in serum proteins: A new potential indicator to distinguish non-diabetic renal diseases from diabetic nephropathy. RSC Adv..

[18] Adua E., Anto E.O., Roberts P., Kantanka O.S., Aboagye E., Wang W. (2018). The potential of N-glycosylation profiles as biomarkers for monitoring the progression of type II diabetes mellitus towards diabetic kidney disease. J. Diabetes Metab. Disord..

[19] Barrios C., Zierer J., Gudelj I., Štambuk J., Ugrina I., Rodríguez E., Soler M.J., Pavić T., Šimurina M., Keser T. (2016). Glycosylation profile of IgG in moderate kidney dysfunction. J. Am. Soc. Nephrol..

[20] Hatakeyama S., Amano M., Tobisawa Y., Yoneyema T., Tsushima M., Hirose K., Yoneyema T., Hashimoto Y., Koie T., Saitoh H. (2013). Serum N-glycan profiling predicts prognosis in patients undergoing hemodialysis. Sci. World J..

[21] Schuett K., Savvaidis A., Maxeiner S., Lysaja K., Jankowski V., Schirmer S.H., Dimković N., Boor P., Kaesler N., Dekker F.W. (2017). Clot structure: A potent mortality risk factor in patients on hemodialysis. J. Am. Soc. Nephrol..

[22] Li J., Hsu H.C., Mountz J.D., Allen J.G. (2018). Unmasking fucosylation: From cell adhesion to immune system regulation and diseases. Cell Chem. Biol..

[23] Poland D.C.W., Schalkwijk C.G., Stehouwer C.D.A., Koeleman C.A.M., van het Hof B., van Dijk W. (2001). Increased α3-fucosylation of α_1_-acid glycoprotein in Type I diabetic patients related to vascular function. Glycoconj. J..

[24] Singh S.S., Heijmans R., Meulen C.K.E., Lieverse A.G., Gornik O., Sijbrands E.J.G., Lauc G., van Hoek M. (2020). Association of the IgG N-glycome with the course of kidney function in type 2 diabetes. BMJ Open Diabetes Res. Care.

[25] Fang M., Kang L., Wang X., Guo X., Wang W., Qin B., Du X., Tang Q., Lin H. (2019). Inhibition of core fucosylation limits progression of diabetic kidney disease. Biochem. Biophys. Res. Commun..

[26] Bermingham M.L., Colombo M., McGurnaghan S.J., Blackbourn L.A.K., Vučković F., Pučić Baković M., Trbojević-Akmačić I., Lauc G., Agakov F., Agakova A.S. (2018). SDRN Type 1 Bioresource Investigators. N-glycan profile and kidney disease in type 1 diabetes. Diabetes Care.

[27] Burkart J. (2004). Metabolic consequences of peritoneal dialysis. Semin. Dial..

[28] Farrell D.H. (2004). Pathophysiologic roles of the fibrinogen gamma chain. Curr. Opin. Hematol..

