# Supplementary material for: Fibrinogen Fucosylation as a Prognostic Marker of End-Stage Renal Disease in Patients on Peritoneal Dialysis
Source: Biomolecules. 2020 Aug 9;10(8):1165. doi: 10.3390/biom10081165 (PMC7466146; doi:10.3390/biom10081165)
Supplement: Supplementary file 1 [file biomolecules-10-01165-s001.pdf]

biomolecules

MDPI

Article

Supplementary Material

Fibrinogen Fucosylation as a Prognostic Marker of End-Stage Renal Disease in Patients on Peritoneal Dialysis

Marko Baralić [1], Nikola Gligorijević [2], Voin Brković [1], Jaroslav Katrlík [3], Lucia Pažitná [3], Miloš Šunderić [2], Goran Miljuš [2], Ana Penezić [2], Zorana Dobrijević [2], Mirjana Laušević [1,4], Olgica Nedić [2] and Dragana Robajac [2,*]

[1] Department of Nephrology, Clinical Centre of Serbia, 11000 Belgrade, Serbia; baralicmarko@yahoo.com (M.B.); voin.brkovic@gmail.com (V.B.); mlausevic@gmail.com (M.L.)
[2] Department of Metabolism, Institute for the Application of Nuclear Energy (INEP), University of Belgrade, 11080 Belgrade, Serbia; nikolag@inep.co.rs (N.G.); milos@inep.co.rs (M.Š.); goranm@inep.co.rs (G.M.); anap@inep.co.rs (A.P.); zorana.dobrijevic@inep.co.rs (Z.D.); olgica@inep.co.rs (O.N.)
[3] Institute of Chemistry, Slovak Academy of Sciences, 84538 Bratislava, Slovakia; katrlik@yahoo.com (J.K.); pazitna.lucia@gmail.com (L.P.)
[4] School of Medicine, University of Belgrade, 11000 Belgrade, Serbia
* Correspondence: draganar@inep.co.rs; Tel.: +381-113-169-058; Fax: +381-112-618-724

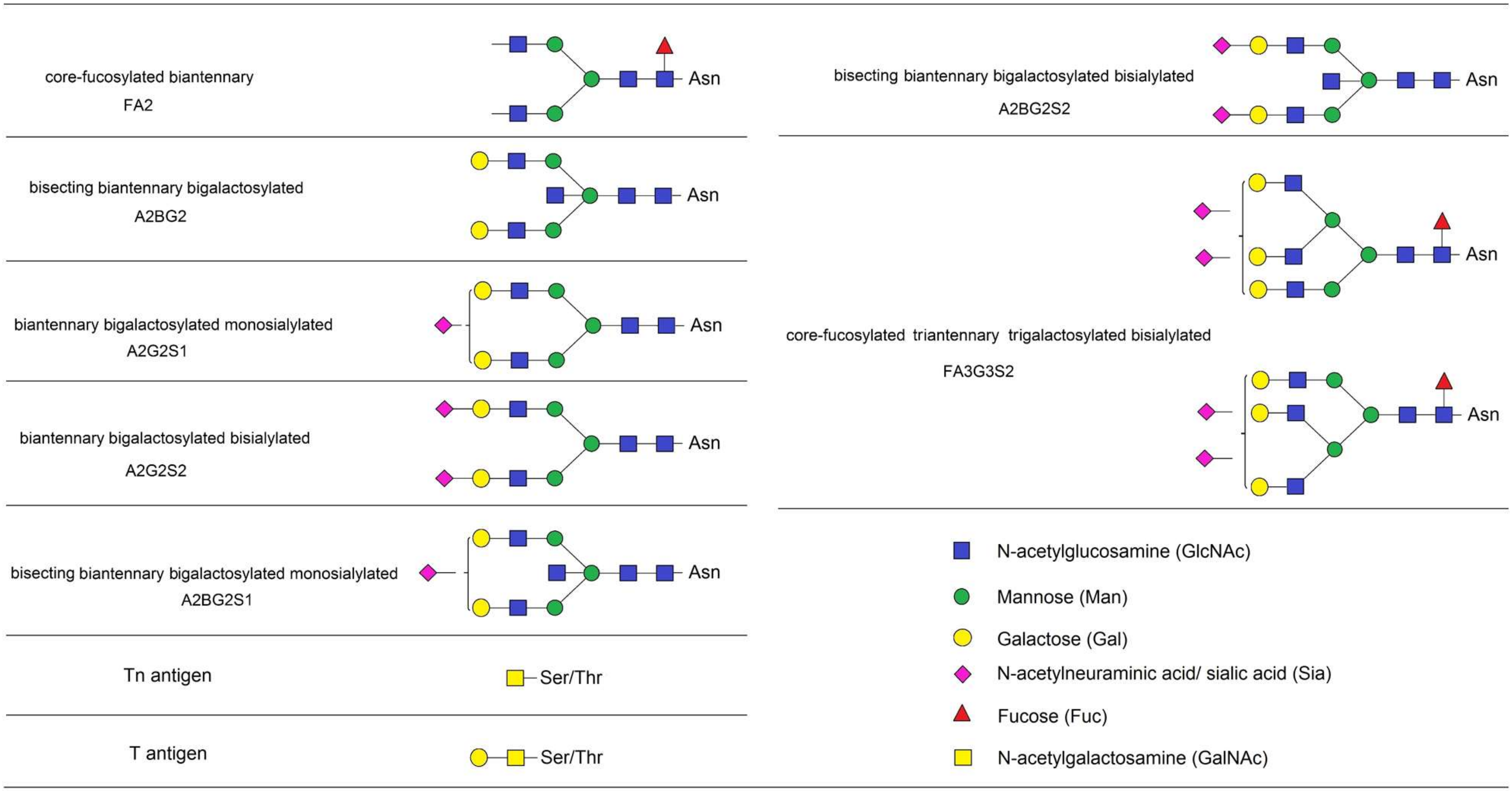

Figure S1. Structures of N- and O-glycans mentioned in the manuscript, with the carbohydrates annotation according to the recommendations of the Consortium for Functional Glycomics (CFG).

© 2020 by the authors. Submitted for possible open access publication under the terms and conditions of the Creative Commons Attribution (CC BY) license (http://creativecommons.org/licenses/by/4.0/).
